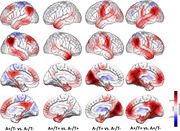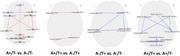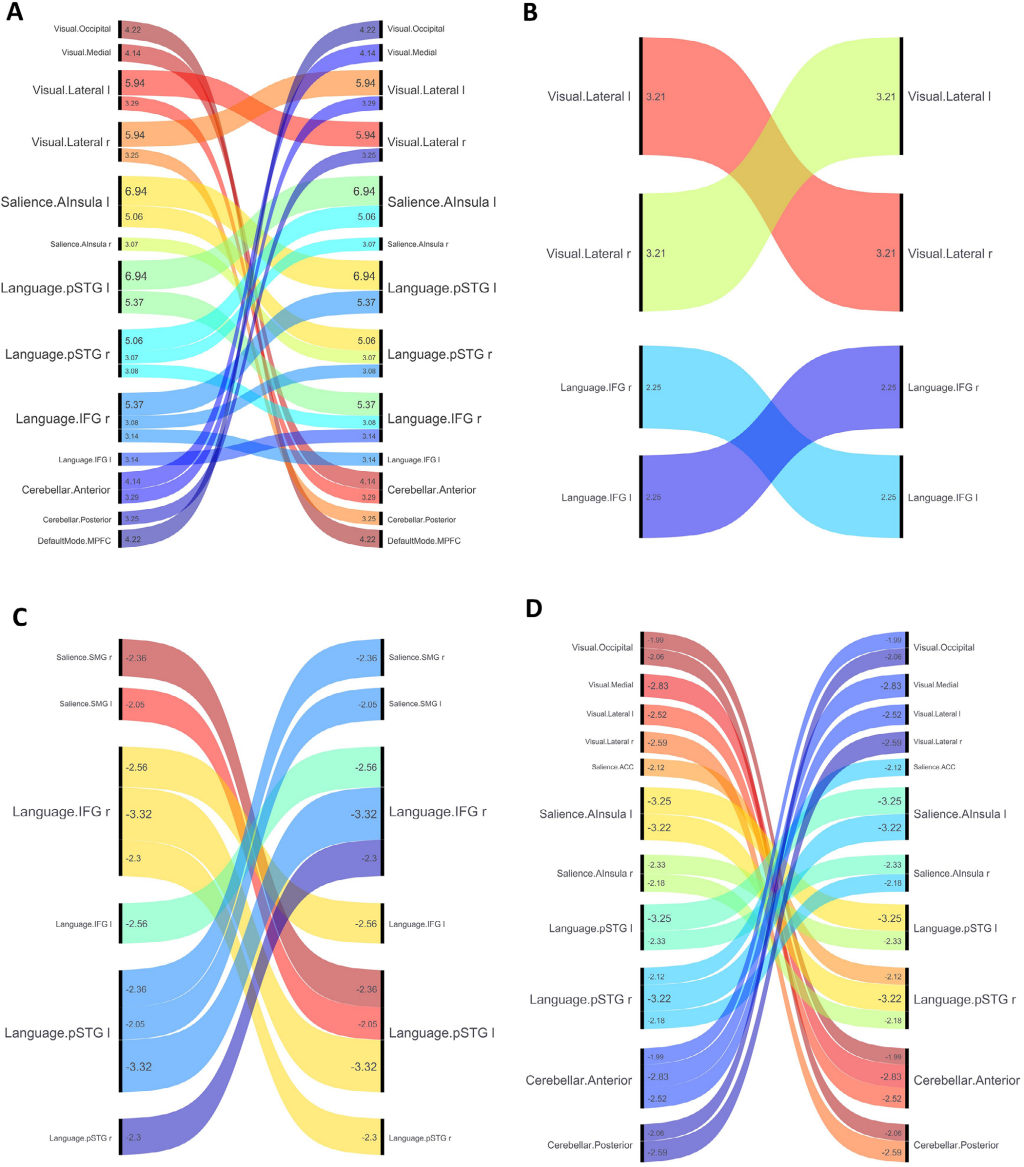# Amyloid‐β deposition speeds up the production of tau tangles through brain networks

**DOI:** 10.1002/alz70856_104805

**Published:** 2026-01-07

**Authors:** Wanwan Guo, Hongda Shao, Yan Zhang, Hairong Zheng, Dong Liang, Jianjun Liu, Lin Liu, Lingyan Zhang, Tengfei Guo, Zhanli Hu

**Affiliations:** ^1^ Longgang Central Hospital of Shenzhen, Shenzhen, Guangdong, China; ^2^ Shenzhen Institute of Advanced Technology, Chinese Academy of Sciences, Shenzhen, Guangdong, China; ^3^ Ren Ji Hospital, Shanghai Jiao Tong University School of Medicine, Shanghai, Shanghai, China; ^4^ Shenzhen Bay Laboratory, Shenzhen, Guangdong, China; ^5^ Tsinghua Shenzhen International Graduate School (SIGS), Tsinghua University, Shenzhen, China; ^6^ Shenzhen Clinical Medical College, Guangzhou University of Chinese Medicine, Shenzhen, Guangdong, China; ^7^ Longgang Clinical Institute of Shantou University Medical College, Shenzhen, Guangdong, China; ^8^ Institute of Biomedical Engineering, Shenzhen Bay Laboratory, Shenzhen, China; ^9^ Peking University Shenzhen Graduate School, Shenzhen, Guangdong, China; ^10^ Key Laboratory of Biomedical Imaging Science and System, Chinese Academy of Sciences, State Key Laboratory of Biomedical Imaging Science and System, Shenzhen, Guangdong, China

## Abstract

**Background:**

Amyloid‐β (Aβ) and neurofibrillary tau deposition are two hallmark pathological proteins of Alzheimer's disease (AD) that accumulate through brain networks and drive cognitive decline. This study investigates whether the functional network abnormalities influence the Aβ‐tau interactions and cognitive impairment in AD, which may provide more insightful perspectives in understanding the neural mechanisms and pathogenesis of AD.

**Method:**

We divided the 190 participants from Shanghai Renji Hospital (68.6 ± 8.4 years, 62% female) into three groups, A‐/T‐ (control group, N = 48), A+/T‐ (*N* = 121), and A+/T+ (*N* = 21), based on established global ^18^F‐AV‐45 amyloid PET thresholds and ^18^F‐PI‐2620 PET (tau PET) thresholds. All subjects underwent ^18^F‐AV‐45 PET, ^18^F‐PI‐2620 PET, resting state functional magnetic resonance imaging (fMRI) and T1‐weighted MRI scans. Functional activity and functional network connectivity were determined using regional homogeneity (ReHo) and functional connectivity (FC) respectively.

**Result:**

Participant demographics and summary descriptive statistics of the cognitive assessments and the PET data analyses are provide Table 1. The health control group (A‐/T‐, n = 48): The average age is 69.9 ± 8.3 years, with 62.5% being female. The mean education level is 10.1 ± 4.0 years. A+/T‐ Group (*n* = 121): The average age is 68.2 ± 8.9 years, with 60.0% being female. The mean education level is 10.4 ± 3.9 years. A+/T+ group(*n* = 21): The average age is 68.3 ± 5.0 years, with 71.4% being female. No significant difference was found for age and years of education across health control group and AD groups. We compared the brain's functional activity among A‐/T‐ group, A+/T‐ group, and A+/T+ group, and we found that functional activity in some brain regions, such as bilateral cerebellum, right insula cortex, right precentral gyrus, right middle frontal gyrus, differed significantly among these forementioned three groups

**Conclusion:**

This study found significant differences in functional activity in brain regions such as the cerebellum, insula cortex, precentral gyrus, and middle frontal gyrus among control, A+/T‐, and A+/T+ AD groups. These results suggest functional network abnormalities may influence Aβ‐tau interactions and contribute to cognitive impairment in AD, shedding light on the neural mechanisms of the disease.